# Phenolic Compounds Removal from Olive Mill Wastewater Using the Composite of Activated Carbon and Copper-Based Metal-Organic Framework

**DOI:** 10.3390/ma16031159

**Published:** 2023-01-29

**Authors:** Muna A. Abu-Dalo, Nathir A. F. Al-Rawashdeh, Moath Almurabi, Jehad Abdelnabi, Abeer Al Bawab

**Affiliations:** 1 Chemistry Department, Faculty of Science and Arts, Jordan University of Science and Technology, Irbid 22110, Jordan; 2 Department of Chemistry and Biomolecular Science, Clarkson University, Potsdam, NY 13699, USA; 3 Department of Chemistry, School of Science, University of Jordan, Amman 11942, Jordan; 4 Hamdi Mango Center for Scientific Research, University of Jordan, Amman 11942, Jordan

**Keywords:** olive mill wastewater, adsorption, total phenolic content, metal-organic framework, granular activated carbon, composite

## Abstract

As the industry of olive oil continues to grow, the management of olive mill wastewater (OMW) by-products has become an area of great interest. While many strategies for processing OMW have been established, more studies are still required to find an effective adsorbent for total phenolic content uptake. Here, we present a composite of a Cu 1,4-benzene dicarboxylate metal-organic framework (Cu (BDC) MOF) and granular activated carbon (GAC) as an adsorbent for total phenolic content removal from OMW. Experimental results demonstrated that the maximum adsorption capacity was 20 mg/g of total phenolic content (TPC) after 4 h. using 2% wt/wt of GAC/Cu (BDC) MOF composite to OMW at optimum conditions (pH of 4.0 and 25 °C). The adsorption of phenolic content onto the GAC/Cu (BDC) MOF composite was described by the Freundlich adsorption and pseudo-second-order reaction. The adsorption reaction was found to be spontaneous and endothermic at 298 K where ΔS° and ΔH° were found to be 0.105 KJ/mol and 25.7 kJ/mol, respectively. While ΔGº value was −5.74 (kJ/mol). The results of this study provide a potential solution for the local and worldwide olive oil industry.

## 1. Introduction

The International Olive Oil Council reported that the yearly production rate of olive oil has grown from roughly 1.5 million tons in 1990 to more than 3.5 tons in 2021 (International Olive Council. 2022). This expansion of the olive oil industry is combined with the generation of 30 million tons of olive mill wastewater (OMW) annually in the Mediterranean basin. In Jordan, approximately 215,000 tons of olives have been processed in 2019 and 169,000 tons in 2020, which generated more than 162,000 m^3^ of OMW in annual seasons of less than one hundred days (October to December) [1], which makes storage in mills impossible. OMW has a high organic and phenolic load with chemical oxygen demand (COD) ranging from 50 to 150 g L^1^, and total phenolic content ranging from 0.5 to 25 g L^−1^ [2]. The management of OMW represents a serious challenge because of its environmental negative impact [3]. OMW show significant polluting properties due to their content of organic substances, and because of their high toxicity toward several biological systems. OMW toxicity has been attributed to its phenolic constituents [4]. Therefore, it is crucial to develop an efficient approach to OMW treatment.

To date, different treatment approaches have been proposed to decrease the OMW environmental effects, such as thermal [5], physical [6], chemical [7], and biological processes [8]. A review was published by Al Bawab 2017 [9] summarizing many conducted studies in the Mediterranean region that investigated different OMW treatment methods, where many chemical, physical, and biological methods have been listed for OMW.

However, coupling the physical process with the chemical process establishes advanced techniques that enhance the quality of OMW [10,11,12,13,14]. On the other hand, membranes [15] and nano photocatalysts [16] methods show good results for treating OMW. Particularly, the adsorption process is considered to be effective technology for OMW treatment applications due to its design simplicity, flexibility, availability, low cost, limited space requirements, and smell-free emissions [17].

Metal-organic frameworks (MOFs), are a rapidly expanding family of crystalline porous material prepared by coordinated metal ions (or clusters) with organic linkers. MOFs are characterized by their high surface area, high porosity, adjustable pore diameters, tunable morphology, and accessible active sites [18,19,20,21,22]. Owing to these impressive properties, MOFs are deemed to be an ideal candidate for adsorption [23]. MOFs were utilized for the adsorption of different chemical substances such as antibiotics pollutants [24], dyes [25], indole and quinoline from a model fuel [26], phenol and p-nitrophenol from aqueous solutions [27]. Nevertheless, the adsorbing capability for total phenolic content from real OMW samples has not been investigated until now by using MOF’s. 

On the other hand, granular activated carbon (GAC) with a high degree of porosity and high surface area has received notable attention for adsorption applications of OMW contaminants [28]. For instance, commercial activated carbon for phenolic compounds [29], and biophenols [30] adsorption was investigated. In Jordan, different methods were used to reduce the OMW impact, and these methods were reviewed by Al Bawab et al., 2018 [13]. Moreover, Odeh et al., 2022 prepared nanocomposite media of (Fe_3_O_4_, FeO(OH)/zeolite via mixing) with GAC for OMW remediation [12]. Activated carbon coated with milk proteins was utilized for phenolic compounds from actual OMW [31].

Additionally, GAC from Jordanian functionalized olive cake with Cu/Cu_2_O/CuO has been employed for total phenolic compounds removal from OMW [11]. Activated carbon obtained by chemical activation of olive pomace and encapsulated in calcium alginate was used for polyphenols uptake from OMW [32].

However, to the best of our knowledge, coupling the GAC with MOF has not been investigated previously for OMW treatment. Although, combining GAC with MOF demonstrated its capability for adsorbing different compounds. For example, GAC/MIL-101(Cr) showed its efficiency for anionic dye removal from aqueous solutions [33]. (Fe-BDC) MOF impregnation within the GAC enhanced the adsorption efficiency of GAC toward Cr (VI) adsorption [34]. Moreover, zirconium MOFs integrated into GAC were able to capture an organo-phosphate nerve agent simulant efficiently [35]. 

This study aims to examine the viability of delivering highly effective GAC/MOF composite adsorbent for scavenging phenols and polyphenols from OMW. Copper-based MOF was selected due to its water stability, high surface area, and high porosity [36]. The newly prepared adsorbent product can be employed by olive-mill owners to reduce the environmental effects of OMW. Moreover, this study provides valuable insight into the effect of coupling the MOF with GAC on enhancing the adsorption properties of the latter, which is critical for the olive oil industry.

## 2. Experimental Part

### 2.1. Materials

Granular activated carbon (GAC) (A Kuraray company, Feluy, Belgium), OMW (pH of 4.0, and TPC of 440 mg/L, Jarash, Jordan), Folin reagent (Sigma-Aldrich, St. Louis, MO, USA), Sodium carbonate 99.8% (Ficher chemical company, Hampton, New Hampshire. USA), Ethyl acetate 99.9% (Carlo Erba reagents, Chau. du Vexin, 27100 Val-de-Reuil, France), n-Hexane 95% (Anavator performance materials, Gliwice, Poland), NaCl 99.9% (Loba Chemie PVT, Mumbai, India), Sodium hydroxide 99.9% (VER International, Foster City, CA, USA), HCl 37% (Scharlau company, Barcelona, Spain), terephthalic acid (H_2_BDC, Merck Schuchardt OHG, Hohenbrunn, Germany), Nitric acid 70% (biosolve Chimie SARL, Dieuze, France), Copper nitrate (Scharlau company, Barcelona, Spain), Gallic acid (99%, Xilong, China), methanol 99.9% (HPLC grade, ACS, Point Pleasant, NJ, USA).

### 2.2. Instruments and Characterization

Transmission FTIR measurements were performed on a Bruker Tensor-27 spectrometer to identify the functional groups on the surface of the adsorbents. The XRD for GAC/Cu(BDC) MOF composite and the parent GAC was performed on an Ultima IV (Rigaku, Tokyo, Japan) with continuous scanning mode, 40 kV/40 mA X-ray, and 4.0 deg/min speed to verify the crystalline nature of the prepared media. The X-ray fluorescence (XRF) (Rigaku, Nexqc+, Austin, TX, USA) technique was used for chemical analysis. CHNS elemental Analyzer (EA3000 Euro Vector, Pavia, Italy) was used for elemental analysis. Quanta FEG 450 Scanning Electron microscope (FEI, Hillsboro, OR, USA) was used to determine surface morphology. The thermal stability of the media was investigated by using (TGA, Netzsch TG 209 F1 Iris). Additionally, the specific surface area and porosity were calculated by using (N_2_ adsorption–desorption isotherms at 77.4 K using a surface area analyzer (Quantachrome Corporation, 360 Engineering, Golden, CO, USA). The pH drift method was used to determine the point of zero charges (pH_PZC_) and Folin–Ciocalteau colorimetric was used for total phenolic content (TPC) determination according to previously reported procedures [11]. The GAC/ Cu(BDC) MOF composite after adsorption (GAC/Cu(BDC) MOF composite*) was characterized to support adsorption mechanism understanding.

### 2.3. Preparation of GAC/Cu(BDC) MOF Composite Adsorbent 

The GAC/Cu(BDC) MOF composite was prepared through the hydrothermal method according to the previously reported method with minor changes [34]. Briefly, 0.8 g of GAC was added to 20 mL of (1 mmol) sodium terephthalate solution (which was prepared by adding 2 mmol of sodium hydroxide to 1 mmol of terephthalic acid) and stirred for 2 h at 45 °C. Then 20 mL of (1 mmol) copper nitrate solution was added to the reaction vessel and stirred for another 2 h. The mixture was refluxed overnight, the final product was washed with deionized water after filtration and then dried at 90 °C for 60 min. 

### 2.4. Adsorption of OMW

Experimentally GAC/Cu(BDC) MOF composite was added to OMW and then the mixture was shaken to reach equilibrium. The OMW was collected by centrifuge at 5000 rpm for 15 min and filtered for total phenol analysis. The maximum adsorption capacity of the GAC/MOF composite was achieved by studying different adsorption conditions (equilibrium time (0.5, 1, 2, 4, 24, and 48 h), adsorbent dose (1%, 2%, 3%, and 4% by weight), temperature (278, 298, and 303 K), and pH (2, 4, 6, and 8)). The removal efficiency % was evaluated using Equation (1).

(1) $$\mathrm{Removal}\;\%=\frac{C_{0}-C_{e}}{C_{0}}*100\%\;$$

where $C_{0}$ is the initial TPC concentration, and $C_{e\;}$ is the equilibrium TPC concentration.

### 2.5. Adsorption Isotherms

At constant temperature, adsorbent and adsorbate interaction processes may be described using adsorption isotherm models which can provide mechanism information of the adsorption process. Therefore, one of the most important ways to estimate the adsorption processes of distinct adsorption systems is to comprehend the modeling of the equilibrium data. The q_t_, and q_e_ are the amount of TPC collected at any time, equilibrium time, respectively, per unit mass GAC/Cu (BDC) MOF composite in (mg/g) is evaluated using Equations (2) and (3). Langmuir Equation (4), and Freundlich Equation (5) models were studied in this work [37]. The linear forms of the Langmuir equation and Freundlich equation are shown in Equations (6) and (7), respectively.

(2) $$q_{t}\;=\;\;\frac{\left(C_{0}-C_{t}\right)V}{m}$$

(3) $$q_{e}\;=\;\frac{\left(C_{0}-C_{e}\right)V}{m}$$

where C_t_ is the TPC at any time, C_e_ is the TPC concentration at equilibrium, C_0_ is the initial TPC concentration in mg/L, V is the sample volume in (L), and (m) is the mass of GAC/Cu(BDC) MOF composite in (g).

(4) $$q\;=\;\frac{{Q\;K}_{L}{\;C}_{e}}{1+\;\left(a_{L}{\;C}_{e}\right)}$$

R_L_ = 1/(1 + bQ_m_) (5)

ln(qe) = ln(k_F_) + 1/n ln(C_e_) (6)

(7) $$\frac{C_{e}}{q_{e}}=\frac{1}{{\mathrm{bQ}}_{m}}+\frac{1}{Q_{m}}C_{e}$$

where q_e_ is equilibrium concentration (mg/g), Ce equilibrium concentration (mg/dm^3^), and Q is an energy term and, in most cases, equal unity. K_L_ (dm^3^/g) and a_L_ (dm^3^/mg) are the Langmuir constants. R_L_ dimensionless constant separation factor and b is the equilibrium constant or Langmuir constant related to the affinity of binding sites (L/g), and Q_m_ represents a particle limiting adsorption capacity [38]. while K_F_ is the Freundlich constant (mg/g)/(dm^3^/mg)^n^ and n is the heterogeneity factor. K_F_ and 1/n were evaluated from a plot of ln qe versus ln C_e_ in Equation (6) which gives a linear plot, the intercept, and slope representing K_F_ and 1/n, respectively. while Q_m_ and b were from the slope and intercept of the linear plot of C_e_/q_e_ versus C_e_ in Equation (7).

### 2.6. Kinetic Modeling

The pseudo-first-order in Equation (8), and pseudo-second-order model in Equation (9), were studied for adsorption kinetics modeling.

ln (q_e_ − q_t_) = ln q_e_ − k_1_ t (8)

(9) $$\frac{t}{q_{t}}=\frac{1}{k_{2}{\;q}_{e}}+\frac{t}{q_{e}}$$

where k_1_ (h^−1^), and k_2_ (g/mg·h), are the equilibrium rate constants of first-order, and second-order, adsorption reactions, respectively, and t is the reaction time in a minute.

### 2.7. Thermodynamic Study

Thermodynamic parameters Gibbs free energy (ΔG°), enthalpy (ΔH°), and entropy (ΔS°) of adsorption from solutions provide a great deal of information concerning the type and mechanism of the adsorption process. Thermodynamic parameters for TPC adsorption on GAC/Cu (BDC) composite were evaluated at 25 °C by the following Equations (10) and (11) [39]. A negative value of Gibbs free energy shows the spontaneous nature and viability of the adsorption process. The ΔH° and ΔS° values were evaluated from the slope and intercept of the linear plot of (ln K) versus (1/T) of Equation (11).

(10) ΔG° = −RT ln K

(11) ln K = (ΔS°)/R − (ΔH°)/RT 

where the equilibrium constant calculated by Equation (12).

(12) $$k=\frac{c_{0}-c_{e}}{c_{e}}\;$$

## 3. Results and Discussions

### 3.1. Characterization of the Media

The elemental analyzer and X-ray fluorescence (XRF) were used to study the elemental composition of parent GAC and GAC/Cu (BDC) MOF composite to identify the structural changes; these results are represented in Table 1. XRF results show that parent GAC contains 20.7% of silicon and 20.1% of aluminum impurities that could be incorporated into the structure from the precursor used for GAC synthesis, which reduced to 9.03%, and 9.23%, respectively, after composition, this reduction could be suggested for substitution with copper. After GAC/Cu (BDC) MOF composite preparation, the copper ratio increased from 0% to 69.9% and the mass of carbon was reduced by 18%, as observed in the elemental analyzer results. The presence of copper is evidence of successful composite synthesis [40].

**Figure 1:**
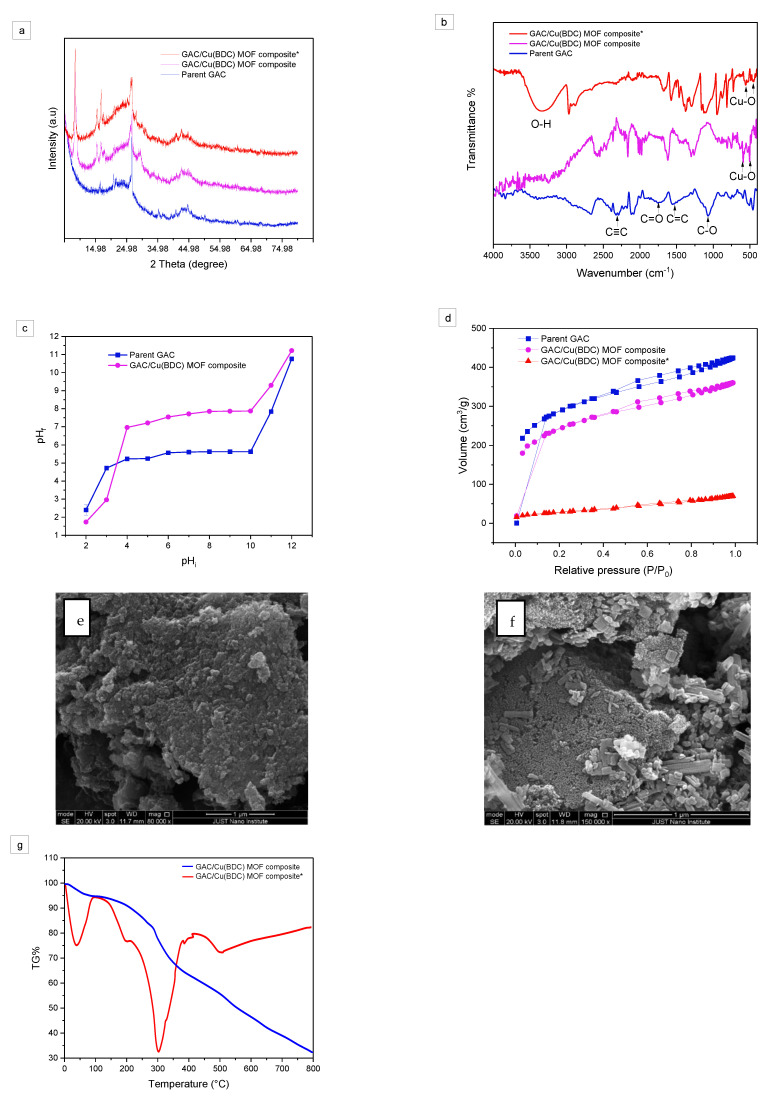
Characterization of Parent GAC, GAC/Cu(BDC) MOF composite, and GAC/Cu(BDC) MOF composite* by (**a**) XRD, (**b**) FTIR, (**c**) the point of zero charges (pHpzc), (**d**) N_2_ adsorption-desorption isotherms at 77.4 K, (**e**) SEM images (parent GAC), (**f**) SEM images (GAC/Cu(BDC) MOF composite), and (**g**) TGA.

The X-ray diffraction (XRD) patterns of the parent GAC, and the composite before and after adsorption of total phenol are shown in Figure 1a. For the parent GAC, two main peaks were observed at 2θ of 42.7°, and 26.6° with miller indices of (100), and (002), respectively. The minor peak at 28.4° can be assigned to the (111) plane of silicon impurity detected by XRF [11]. On the other hand, the XRD pattern of the GAC/Cu (BDC)MOF composite shows a high-intensity sharp peak at 2θ of and 8.6°(001), and two small peaks at 2θ of 15.7°(420), and 16.6°(422), which indicates the successful formation of crystalline Cu(BDC) MOF on the GAC surface [41]. After the OMW adsorption, the XRD pattern of (GAC/Cu(BDC) MOF composite*) still shows the same characteristic peaks of the GAC/Cu (BDC) MOF composite, indicating that the adsorption of total phenols did not affect the structure and the crystallinity of the composite. The crystal size (D_avg_ around 11.4 nm) for GAC/Cu (BDC) MOF composite was calculated by using Scherrer Equation (13) [42].

(D = Kλ/β. cosθ) (13)

in which;

K: Scherrer’s constant (0.9)
D: the crystal size
λ: the wavelength of X-ray (1.5 nm)
β: the width of the peak in the middle of the height
θ: the angle between the X-ray and the particle

To estimate the functional groups on the surface of the parent GAC, GAC/Cu (BDC) MOF composite, the Fourier transform infrared (FTIR) analysis was performed and represented in Figure 1b. The FTIR spectra of parent GAC shows peaks at 2838–2960 cm^−1^, 3000–3075 cm^−1^, and 3300–3310 cm^−1^ proposing the existence of sp^3^, sp^2^, and sp C-H stretching, respectively. Peaks at 1560 cm^−1^, around 2300 cm^−1^, and around 2000 cm^−1^ were observed, suggesting the presence of C=C, C≡C, and C=C=C functional groups. The peak at 3,390 cm^−1^ is assigned to O-H stretching. Correspondingly, C-O and C=O stretching peaks were observed at 1017 cm^−1^ and 1718–1863 cm^−1^ [43]. After GAC/Cu-BDC MOF composite formation, the Cu-O stretching vibration peaks were observed at 500 cm^−1^ and 586 cm^−1^ [40,44]. The peaks around 831 cm^−1^ and 1620 cm^−1^ could be suggested as the presence of C-H vibration, and C=C stretching of the benzene ring in the terephthalic acid ligand [44,45]. On the other hand, the FTIR of GAC/Cu(BDC) MOF composite* shows peaks at 3370 cm^−1^, which could be suggested to be the OH group from water adsorbed and peaks at 2974 cm^−1^, 2877 cm^−1^ and at 1371 cm^−1^ correspond to the CH_2_ symmetric frequency (CH_2_ stretching frequency), and OH phenolic group, respectively [46]. Moreover, we can notice that Cu-O stretching vibration peaks were shifted to around 489 cm^−1^ and 555 cm^−1^ which demonstrates the contribution of the Cu-O functional group in the TPC uptake [47], which also indicates that the GAC/Cu (BDC) MOF composite has good stability in OMW.

To understand the adsorption behavior of adsorbents and the capability for electrostatic interaction with dissolved TPC, the point of zero charges (pH_pzc_) was measured and presented in Figure 1c. The pH_pzc_ for parent GAC is 7.8, which is compatible with the previously reported value [48]. However, the pH_PZC_ value GAC/Cu(BDC)MOF composite was reduced to 5.6, due to the presence of carboxylic acid functional group (pka = 4.1) in the Cu(BDC) MOF. These results collectively imply that the surfaces of GAC/Cu(BDC) MOF composites are positively charged at a pH lower than 5.6 and negatively charged above that value. 

The surface area of the parent GAC, GAC/Cu(BDC) MOF composite, and GAC/Cu(BDC) MOF composite* are depicted in Figure 1d, which shows that the N_2_ adsorption-desorption isotherm is of type IV according to the IPUAC classification, having H3 type hysteresis loop in the latter half part (P/P0 is 0.5~1.0), indicating that the product has a typical mesoporous structure [49,50]. Loading the Cu(BDC) MOF onto the GAC decreased the specific surface area of the parent GAC from 949.689 to 805.597 m^2^/g as a consequence of Cu(BDC) MOF coating the surfaces and filling the pores of the GAC. Additionally, the porous volume dropped from 0.65547 to 0.55659 cm^3^/g. On the other hand, the pore size of the parent GAC and GAC/Cu(BDC) MOF composite are 27.608 Å and 27.636 Å, respectively. However, the impregnation of Cu(BDC) MOF within the pores of parent GAC considerably improves the specific adsorption of TPC which could be due to an increase in active sites with the presence of copper metal and carboxyl group [51]. After TPC adsorption, the surface area and porous volume reduced to 102.5 m^2^/g and 0.1077 cm^3^/g, respectively, as a result of the filling of pores with TPC. An identical result was observed earlier in the adsorption of toluene onto porous Cu–BDC@OAC composite [40]. 

Scanning electron microscopy (SEM) characterization was carried out for the parent GAC, and GAC/Cu(BDC) MOF composite to highlight the morphological changes that happened to the media surfaces and the distribution of components before and after the formation of the composite. As illustrated in Figure 1e, GAC has an amorphous phase, while GAC/ Cu (BDC) MOF composite in Figure 1f has rod and long bar-likes crystals of Cu (BDC) MOF distributed on the GAC surface.

In order to systematically engineer the properties of crystalline and amorphous metal-organic frameworks (MOFs) towards practical application, a detailed understanding of their high-temperature behavior is required. The thermal stability of MOF-composites is affected by the materials used, the composite, and the amount of composite added. Therefore, in this study, in order to know whether the synthesized GAC/Cu (BDC) MOF composite could be used as new materials it is necessary to study their thermal stability. Thermogravimetric analysis (TGA) was used to determine the thermal stability of the synthesized GAC/Cu(BDC) MOF composite and GAC/Cu(BDC) MOF composite*, as well as the quantify of deterioration of Cu(BDC) MOF that could leak in water, as shown in Figure 1g. Three distinct areas had the most noticeable weight decrease. For GAC/Cu (BDC) MOF composite and GAC/Cu (BDC) MOF composite*, the initial loss occurred between 30 °C and 290 °C; this stage indicates the water loss, whether it is chemically adsorbed water or physically adsorbed water [52]. The primary deterioration of Cu (BDC) MOF is represented by around 12% weight loss between 300 °C and 390 °C during the second loss [53,54]. The production of CuO as a byproduct of the deterioration and combustion of organic and inorganic is represented by the third stage, which takes place above 440 °C [55,56]. This result demonstrates the thermal stability of the GAC/Cu(BDC) MOF composite in OMW, which revealed that the synthesized GAC/Cu(BDC) MOF composite could sustain high temperatures up to 300 °C.

### 3.2. Adsorption Evaluation

The adsorption behavior of the GAC/Cu(BDC) MOF composite was evaluated to find the best adsorption condition including adsorbent dose, temperature, contact time, and pH to achieve maximum total phenolic compounds (TPC) uptake from Jordanian OMW (pH of 4.0 and TPC of 440 mg/L). The adsorption evaluation tests were investigated with no physical or chemical pretreatment process of OMW. Moreover, kinetic, thermodynamic, and isotherm adsorption was evaluated under the optimum adsorption conditions. 

**Figure 2:**
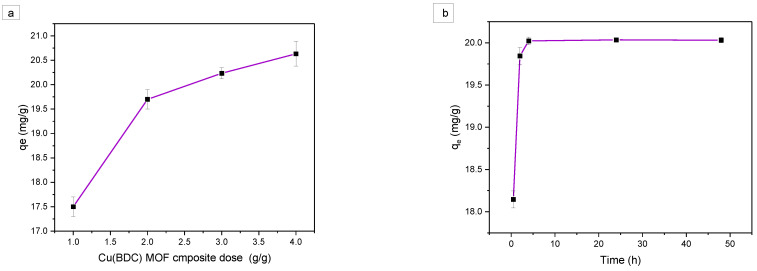

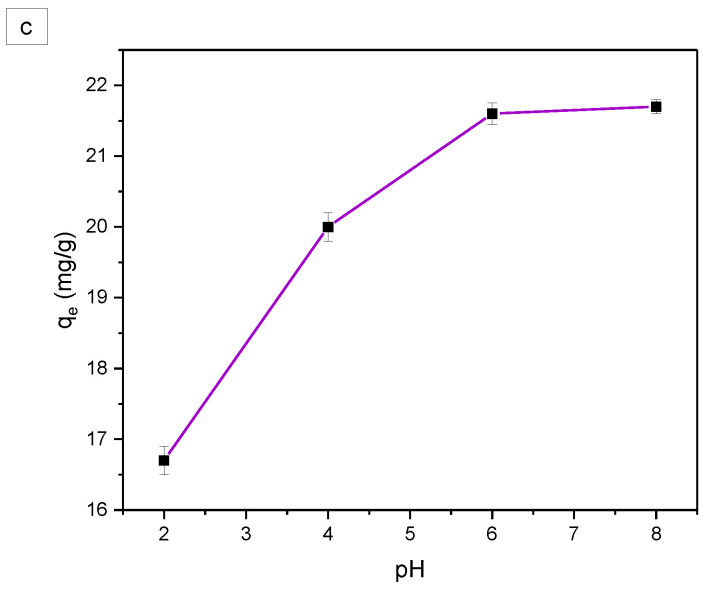
The effect of: (**a**) the adsorbent dose on the adsorption process at a temperature of 298 K, pH 4.0, and initial TPC of 440 mg/L; (**b**) the equilibrium time of the adsorption process at a temperature of 298 K, pH 4.0, 2% by weight adsorbent dose, and (**c**) the OMW pH on the TPC uptake using 2% by weight adsorbent dose, 440 mg/L initial TPC concentration, at a temperature of 298 K, and a contact time of 4 h.

The effect of GAC/Cu(BDC) MOF composite dose on TPC removal for an initial concentration of 440 mg/L, a temperature of 298 K, and a pH of 4.0 was investigated by varying the adsorbent dose from 1% to 4% with respect to OMW wt/wt. The results represented in Figure 2a show that increasing the dose of the GAC/Cu(BDC) MOF increased the equilibrium concentration (q_e_ = mg/g) until reached maximum capacity, which could be attributed to an increase in certain active sites on the adsorbent [57]. However, no significant adsorption enhancement occurred by increasing the dose from 2% to 4% which could be attributed to the blocking of certain active sites on the adsorbent surface because of partial aggregation of the adsorbent particles at high concentrations, thus, decreasing in available surface area for TPC uptake, so a 2% adsorbent dose was selected for further investigations [58]. 

The influence of the contact time on the adsorption process was investigated using a 2% wt./wt. of GAC/Cu (BDC) MOF composite to OMW at a temperature of 298 K, and pH 4.0. Figure 2b has shown that as contact duration is extended, the adsorption efficiency initially increases and then gradually stabilizes and reaches equilibrium after 4 h with a maximum equilibrium capacity of 20.01 mg/g. This might be due to the availability of active binding sites for adsorption. Phenol removal within adsorption has been investigated in many studies (Table 2). In this study, the synthesized GAC/Cu(BDC) MOF composite displayed better adsorption capacity towards phenol, compared to other used GAC/adsorbents under the studied conditions, with short contact time (the contact time in this study is 4 h).

The pH has a significant role in TPC adsorption from OMW (Yousef and El-Eswed, 2009). So, it is important to evaluate the best pH value to reach the maximum TPC removal, and to understand the adsorption mechanism of TPC on the adsorbent surface. Some phenolic compounds in water have pKa’s around 4 [41,66]. At pH lower than the pKa, phenolic compounds remain in a protonated form, while at pH values higher than pKa, phenolic compounds dissociate into anionic forms [38]. The pH was tested at pH of 2.0, 4.0, 6.0, and 8.0 with an adsorbent dose of 2% by weight, an initial TPC concentration of 440 mg/L, a temperature of 298 K, and a contact time of 4 h. Figure 2c represents the pH effect on TPC adsorption which indicates an increase in q_e_ from pH 2 up to pH 6 reaching a plateau at pH 6. These results could be attributed to the increase in electrostatic interactions between negatively charged TPC and positively charged GAC/Cu(BDC) MOF composite surface at pH ≤ pH_pzc_ (pH_pzc_ = 5.6). At pH ˃ pH_pzc_, GAC/Cu(BDC) MOF composite surface will be negatively charged. The repulsion between TPC and GAC/Cu(BDC) MOF composite surface will arise, which is adsorbed with more difficulty on GAC/Cu(BDC) MOF composite to negative charge. Since the adsorption showed high efficiency at a pH of 6–8, the repulsion between the phenolate anions and the negatively charged GAC/Cu(BDC) MOF composite surface is not a limiting factor for the adsorption process. High efficiency at a pH of 6–8 can be explained by the complexation reaction between phenolate anions and copper metal on the surface of the adsorbent where the electrons transfer from phenolate to the empty d-orbitals of copper, this result is compatible with the result reported by when they studied the effect of pH on the adsorption of phenol and chlorophenols onto natural zeolite [67].

### 3.3. Estimation of the Thermodynamic Parameters

**Figure 3:**
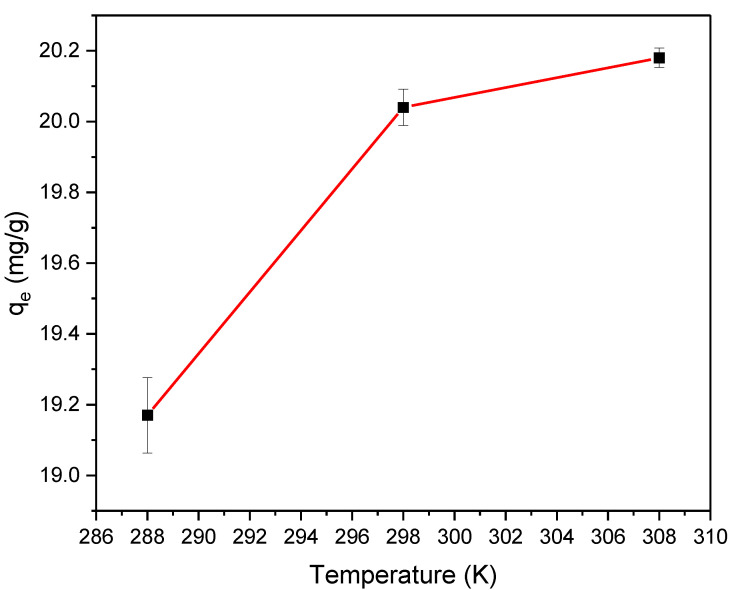
The temperature effect on the TPC removal using a 2% adsorbent dose, pH 4.0, and initial TPC 440 mg/L.

The temperature impact on the adsorption process was investigated using an adsorbent dose of 2% by weight, pH of 4.0, and initial TPC concentration of 440 mg/L at temperatures of 288, 298, and 308. The result represented in Figure 3 demonstrates a direct proportional influence of the temperature on the equilibrium TPC uptake. The highest uptake was at a temperature of 308 K and the lowest TPC uptake was at a temperature of 288 K. As a result, the temperature has a positive influence on TPC adsorption. This behavior suggests that phenol adsorption onto GAC/Cu(BDC) MOF composite is an endothermic process [43], which is confirmed by the thermodynamic study.

To estimate the nature of the adsorption process, the thermodynamic parameters as ΔG°, ΔH° and ΔS° were evaluated. As presented in Table 3, ΔH° was 25.7 kJ/mol which proposes that the adsorption reaction was endothermic and the removal was mainly due to physical adsorption described by electrostatic interaction between the positively charged adsorbent molecules and the negatively charged phenols, as the magnitude of (ΔH°) is lower than 40 kJ/mol [68]. Assuming that ΔH° and ΔS° are temperature independent, the value of ΔG was found to be −4.54, −5.74, and −6.08 (kJ/mol) at 288, 398, and 308 K, respectively. The negative sign of the ΔG indicates that the adsorption process was spontaneous which confirms the practicability of the process. The same result was reported upon using activated carbon derived from Jordanian olive cake and functionalized with Cu/Cu_2_O/CuO for the adsorption of TPC from OMW [11].

### 3.4. Modeling the Reaction Kinetics

**Figure 4:**
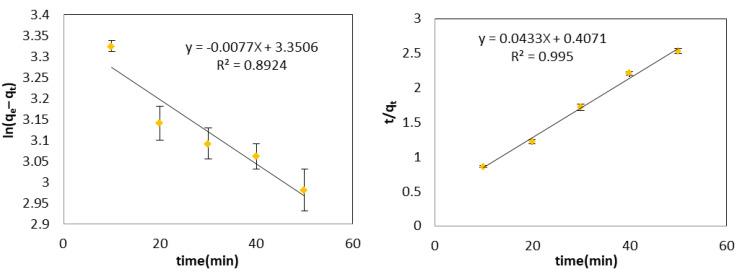
Pseudo-first (**left**) and second (**right**)-order models at pH = 4.0, temperature of 298 K, and A 2% by weight dosage. Error bars represent the standard deviation of triplicate measurements.

Plotting the kinetic data in several reaction models, including pseudo-first-order and pseudo-second-order, allowed for the identification of the reaction order and the best-matched model. The validity of the pseudo-first-order models and pseudo-second-order was examined using the plots of ln(q_e_ − q_t_) and t/q_t_ vs. time, respectively. The adsorption reaction kinetics data may be best described by a pseudo-second-order reaction model, according to the straight lines that were reached in the pseudo-second-order reaction model (R^2^ = 0.995), which was deemed to be the most acceptable as shown in Figure 4. However, the pseudo-first-order model did not show a significant degree of linearity (R^2^ = 0.892). The rate constants for the TPC uptake of the pseudo-first-order model, and pseudo-second-order model were 0.0077 h^−1^ and 0.11 mg/g·h, respectively, which are analogous to previously reported values [69].

### 3.5. Adsorption Isotherm

**Figure 5:**
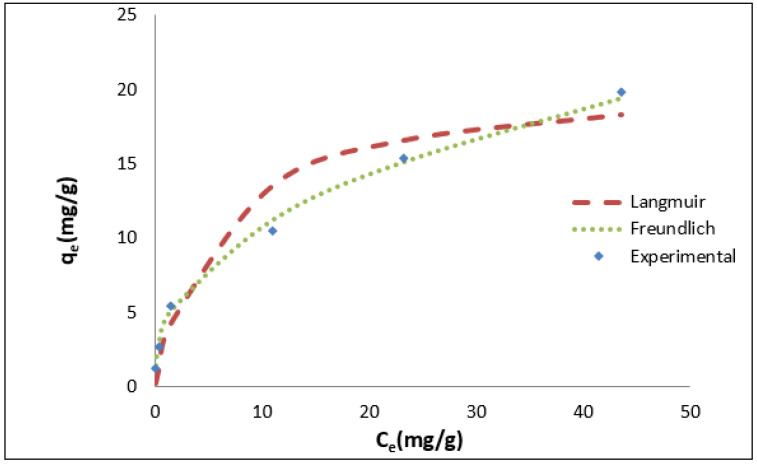
Isothermal adsorption of TPC by GAC/Cu(BDC) MOFcomposite at a temperature of 298 k, initial TPC 440 (mg/L), pH = 4.0, 2% by weight dosage, and 4 h contact time.

As shown in Figure 5, the equilibrium data for the adsorption of TPC by GAC/Cu(BDC) was fitted to the linear form of the Langmuir and Freundlich relations. Table 4 provides the values of the estimated parameters and correlation coefficients (R^2^) at a temperature of 298 °C. The Langmuir adsorption isotherm suggested that there is a limited number of binding sites which distributed homogeneously over the surface of the adsorbent. These sites have the same ability for the adsorption of adsorbate in a monolayer without interaction between adsorbate molecules. R_L_ value was found between 0 and 1 indicating that the adsorption of TPC GAC/Cu(BDC) MOF composite is favorable. The Freundlich isotherm model assumes that the concentration of adsorbate molecules on the adsorbent surface has a direct proportion with adsorbate concentration. Moreover, the “n” value of the Freundlich model was found to be greater than 2 and less than 10, indicating a favorable adsorption process, a heterogeneous surface and that the adsorption process functioned under multilayer adsorption [38,70]. Correlation coefficient R^2^ of the Freundlich model appears to best fit the experimental data more than the Langmuir model.

In summary, from the correlation between the characterization and the Freundlich parameters, it is concluded that the adsorption behavior of phenol on GAC/Cu (BDC) MOF composite is controlled. We added a vertical dotted line by the dispersion force between the π-electrons in activated carbons and those in phenol molecules. Furthermore, it may be a combined mechanism that includes π–π interaction, acid–base interaction, electrostatic interaction, hydrogen bond, metal coordination and hydrophobic interaction [71].

## 4. Conclusions

The composite of copper-based metal-organic frameworks and granular-activated carbon (GAC/Cu(BDC))MOF composite was prepared successfully by a simple hydrothermal method. The combination of Cu(BDC) MOF onto the granular activated carbon (GAC) surface led to a lowering of the point of zero charges (pHpzc) value and surface area, and increasing in the adsorption efficiency due enhancement of binding sites as a result of the present of copper metal. The optimum conditions for TPC (440 mg/L) uptake from OMW by GAC/Cu(BDC) MOF composite were 2% wt/wt of GAC/Cu (BDC) MOF composite to OMW, pH of 4.0, at 25 °C, and contact time of 4 h. The adsorption occurs via different mechanisms including electrostatic interaction, and coordination of phenolate ions to a copper center. The adsorption of TPC on composite material could be described by pseudo-second-order and the Freundlich isotherm adsorption model. The adsorption reaction was found to be spontaneous at 298 K ± 10 K. At optimum conditions, the GAC/Cu(BDC) MOF composite can remove about 91% of phenolic compounds from OMW which can help the owner of the mills’ to manage this wastewater.

## Figures and Tables

**Table 1:** Elemental composition of parent GAC and GAC /Cu (BDC) MOF composite. Reported by CHNS elemental analysis and XRF techniques.

Sample Name	CHN Elemental Analyses			XRF		
	N%	C%	H%	Cu%	Al%	Si%
GAC/Cu(BDC) MOF composite	0.55	63.32	1.46	69.9	9.23	9.03
Parent GAC		81.37	1.37		20.1	20.7

**Table 2:** Efficiency of different adsorbents used for phenolic compounds removal from OMW in the literature, compared to adsorbent prepared in this investigation.

Adsorbent	Initial Total Phenolic Compounds Concentration	Removal%	Contact Time	Reference
GAC/Cu(BDC) MOF composite	440 mg/L	91%	4 h	Current research
Activated carbon (purchased from Strem Chemicals (USA))	10.85 g/L	87%	-	[31]
TRI-SBA-15		75%		
TRI-P-10		67%		
Olive pomace	50 mg/L	up to 40%	24 h	[59]
Activated carbon functionalized with Cu/Cu_2_O/CuO	124 mg/L	85%	24 h	[11]
Activated Clay	1190 mg/L	81%	24 h	[60]
Wood char-based powdered activated carbon, PAC, (SigmaeAldrich)	900 mg/L	87%	24 h	[61]
Pomegranate Seed	162.5 mg/L	Up to 92.8%	10 min	[62]
Activted Volcanic Tuff/0.5% wt/wt magnetite NPs	124 mg/L	73%	48 h	[10]
Activated carbon	1190 mg/L	94%	less than 4 h	[63]
Environmentally compatible activated carbon of different particle sizes (Sigma–Aldrich)	4821.5 mg/L	38–40%	2 h	[30]
Powdered carbon normally has median particle diameters between 10 and 50 mm	450 mg/L	96.6%	4 h	[64]
Clay	4.75 g/L	57.4%	36 h	[65]

**Table 3:** Thermodynamic parameters estimated at different temperatures.

TPC (mg/L)	Temperature (K)	K	ΔG (KJ/mol)	ΔH° (KJ/mol)	ΔS° (KJ/mol)
440	288 298 308	4.3 4.8 5.9	−4.54 −5.74 −6.08	25.7	0.105

**Table 4:** Freundlich and Langmuir parameters, and regression coefficients at temperatures for the adsorption of TPC by GAC/Cu(BDC) MOF composite with a 2% by weight dose, a temperature of 298 K, pH of 4.0, and 4 h contact time.

Temperature (K)	Freundlich Isotherm			Langmuir Isotherm			
298	K_F_	n	R^2^	Q_m_	B	R^2^	R_L_
	4.1	2.5	0.996	8.7	2.7	0.941	0.84

## Data Availability

Not applicable.
